# Brain Angiotensin II Type 1 Receptor Blockade Improves Dairy Blood Pressure Variability via Sympathoinhibition in Hypertensive Rats

**DOI:** 10.1155/2015/759629

**Published:** 2015-03-30

**Authors:** Takuya Kishi, Yoshitaka Hirooka, Kenji Sunagawa

**Affiliations:** ^1^Department of Advanced Therapeutics for Cardiovascular Diseases, Kyushu University Graduate School of Medical Sciences, 3-1-1 Maidashi, Higashi-ku, Fukuoka 812-8582, Japan; ^2^Department of Advanced Cardiovascular Regulation and Therapeutics, Kyushu University Graduate School of Medical Sciences, 3-1-1 Maidashi, Higashi-ku, Fukuoka 812-8582, Japan; ^3^Department of Cardiovascular Medicine, Kyushu University Graduate School of Medical Sciences, 3-1-1 Maidashi, Higashi-ku, Fukuoka 812-8582, Japan

## Abstract

Abnormal blood pressure (BP) elevation in early morning is known to cause cardiovascular events. Previous studies have suggested that one of the reasons in abnormal dairy BP variability is sympathoexcitation. We have demonstrated that brain angiotensin II type 1 receptor (AT_1_R) causes sympathoexcitation. The aim of the present study was to investigate whether central AT_1_R blockade attenuates the excess BP elevation in rest-to-active phase in hypertensive rats or not. Stroke-prone spontaneously hypertensive rats (SHRSP) were treated with intracerebroventricular infusion (ICV) of AT_1_R receptor blocker (ARB), oral administration of hydralazine (HYD), or ICV of vehicle (VEH). Telemetric averaged mean BP (MBP) was measured at early morning (EM), after morning (AM), and night (NT). At EM, MBP was significantly lower in ARB to a greater extent than in HYD compared to VEH, though MBP at AM was the same in ARB and HYD. At NT, MBP was also significantly lower in ARB than in HYD. These results in MBP were compatible to those in sympathoexcitation and suggest that central AT_1_R blockade attenuates excess BP elevation in early active phase and continuous BP elevation during rest phase independent of depressor response in hypertensive rats.

## 1. Introduction

Hypertension is established as a major risk factor for cardiovascular disease, and antihypertensive treatments are necessary to prevent the cardiovascular events [1]. We have already various and effective antihypertensive agents. However, it is also true that our antihypertensive treatments could not achieve the optimal blood pressure levels [2, 3]. Among the unmet prevention for hypertensive cardiovascular events, early morning blood pressure elevation is known to be associated with cardiovascular events [4, 5]. Recent several studies have suggested that morning surge should be a crucial target of the treatments for hypertension [6–8]. There are so much various factors to cause morning surge [4, 5], and, among the factors, sympathoexcitation and baroreflex dysfunction are closely associated with blood pressure elevation in the early morning [9].

Sympathetic nerve activity is mediated by brain, especially by rostral ventrolateral medulla (RVLM) known as vasomotor center [10, 11]. In the aspects of sympathoexcitation, we and other investigators have demonstrated that brain oxidative stress in the RVLM causes sympathoexcitation [12–18] and that blockade of angiotensin II type 1 receptor (AT_1_R) in the RVLM decreases blood pressure with sympathoinhibition and improvement of baroreflex sensitivity via reduction of oxidative stress [12, 17, 18]. From these backgrounds, the aim of the present study was to investigate whether central AT_1_R blockade attenuates the excess blood pressure elevation in early active phase (mimicking early morning surge) with sympathoinhibition and improvement of baroreflex in hypertensive rats and if so to determine whether the benefit was independent of depressor response or not.

## 2. Methods

### 2.1. Studies and Animals

The study protocol was reviewed and approved by the Committee on the Ethics of Animal Experiments at the Kyushu University Graduate School of Medical Sciences and conducted according to the Guidelines for Animal Experiments of Kyushu University. Experiments were performed on male stroke-prone spontaneously hypertensive rats (SHRSP) as a hypertensive model with sympathoexcitation (14 to 18 weeks old, SLC Japan, Hamamatsu, Japan). SHRSP were divided into 3 groups, treated with intracerebroventricular infusion (ICV) of AT_1_R receptor blocker (ARB, *n* = 5), treated with oral administration of hydralazine (HYD, *n* = 5), and treated with ICV of vehicle (VEH, *n* = 5) for 2 weeks.

### 2.2. Administration of Drugs

In ARB, losartan (1 mg·kg^−1^·day^−1^) in artificial cerebrospinal fluid (aCSF) was infused at 0.5 *μ*L/h for 14 days by using an osmotic minipump (Alzet 2002, DURECT Corporation, Cupertino, CA) into the right lateral cerebral ventricle of the brain, as described previously [17, 19]. In HYD, hydralazine (100 mg/L) was administered in the drinking water. In VEH, only aCSF was infused at 0.5 *μ*L/h for 14 days by using an osmotic minipump.

### 2.3. Measurements of Mean Blood Pressure and Heart Rate

Mean blood pressure (MBP) and heart rate (HR) were measured using the UA-10 radiotelemetry system (Data Sciences International, Saint Paul, MN, USA) as described previously [12, 17, 18, 20]. Telemetric MBP of 5 minutes three times every one hour was sampled and averaged at the first 2 hours at dark-active (early morning, EM), mid 2 hours at dark-active (after morning, AM), and mid 2 hours at light-rest phase (night, NT).

### 2.4. Measurements of Sympathoexcitation

We assessed sympathoexcitation by spectral analysis using an adaptive autoregressive model to provide power spectra for systolic blood pressure (SBP). The low-frequency power of SBP (integrating the spectra between 0.04 and 0.15 Hz) was computed by MATLAB (MathWorks, USA), and sympathoexcitation is presented as the normalized unit of the low-frequency component of SBP (LFnuSBP), as previously done in our and other studies [17, 20–22].

### 2.5. Assessment of Baroreflex Sensitivity

We assessed baroreflex sensitivity by spontaneous sequence method, as done in our and other previous experiments [17, 20, 23, 24]. In brief, we measured baroreflex sensitivity by using spontaneous sequence method. About 10-minute rest period was obtained in all subjects to allow for stabilization of blood pressure or HR. For analysis of about 5-minute hemodynamic recordings from telemetry system, we selected all sequences of three or more successive heart beats in which there was concordant increase (up sequence) or decrease (down sequence) in arterial systolic blood pressure and peak-to-peak systolic blood pressure interval change. A linear regression was applied to each of the sequences, and an average regression slope was calculated for the sequences. This slope represents the cardiac baroreflex sensitivity. The threshold values for including beat-to-beat systolic blood pressure and its interval changes in a sequence are set at 1 mmHg and 2 milliseconds, respectively.

### 2.6. Statistical Analysis

All values are expressed as the mean ± SEM. An unpaired *t*-test was used to compare the parameters in each group. Differences were considered significant when the *P* value was less than 0.05.

## 3. Results

### 3.1. Mean Blood Pressure and Heart Rate

**Figure 1:**
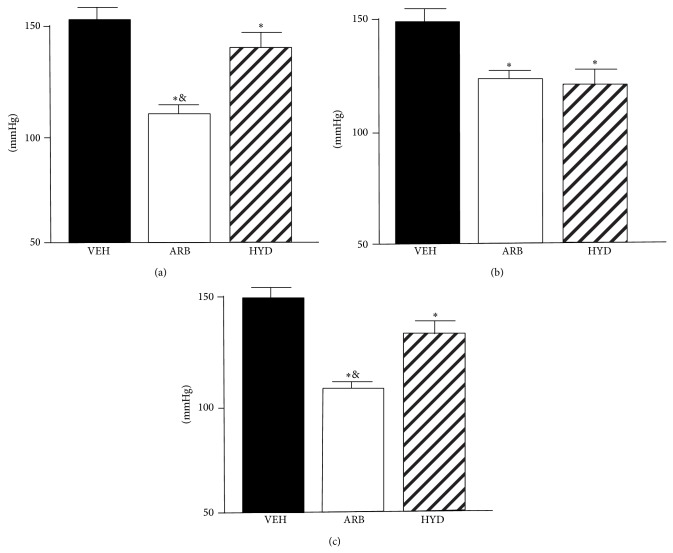
Telemetric averaged mean blood pressure in stroke-prone spontaneously hypertensive rats treated with intracerebroventricular infusion of losartan (ARB, *n* = 5), oral-administered hydralazine (HYD, *n* = 5), and intracerebroventricular infusion of vehicle (VEH, *n* = 5) at early morning (a), after morning (b), and night (c). ^*^ *P* < 0.05 versus VEH, ^&^ *P* < 0.05 in ARB versus HYD.

At EM, MBP was significantly lower in ARB to a greater extent than in HYD compared to VEH, though MBP at AM was the same in ARB and HYD (Figure 1). At NT, MBP was also significantly lower in ARB than in HYD (Figure 1).

**Figure 2:**
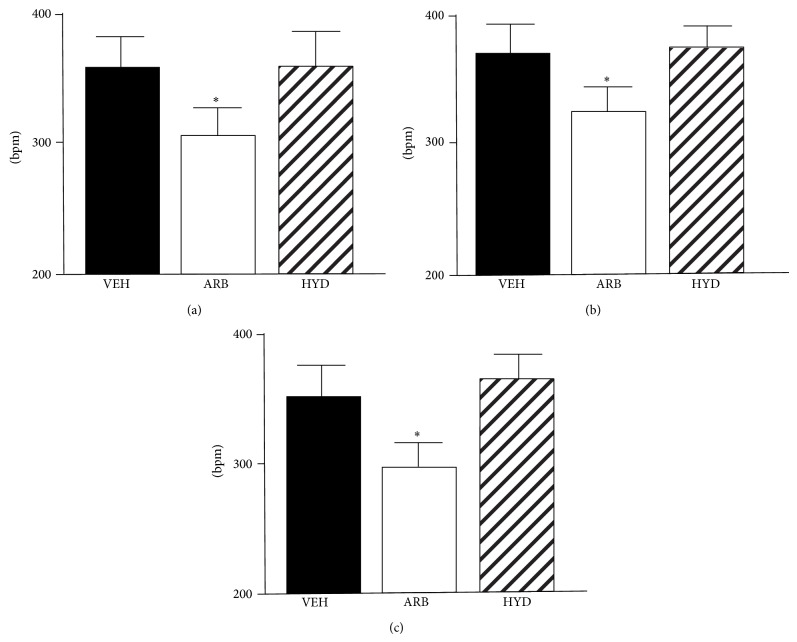
Telemetric averaged heart rate in stroke-prone spontaneously hypertensive rats treated with intracerebroventricular infusion of losartan (ARB, *n* = 5), oral-administered hydralazine (HYD, *n* = 5), and intracerebroventricular infusion of vehicle (VEH, *n* = 5) at early morning (a), after morning (b), and night (c). ^*^ *P* < 0.05 versus VEH.

Throughout a day, HR was significantly lower in ARB than in HYD and VEH (Figure 2).

### 3.2. Sympathoexcitation

**Figure 3:**
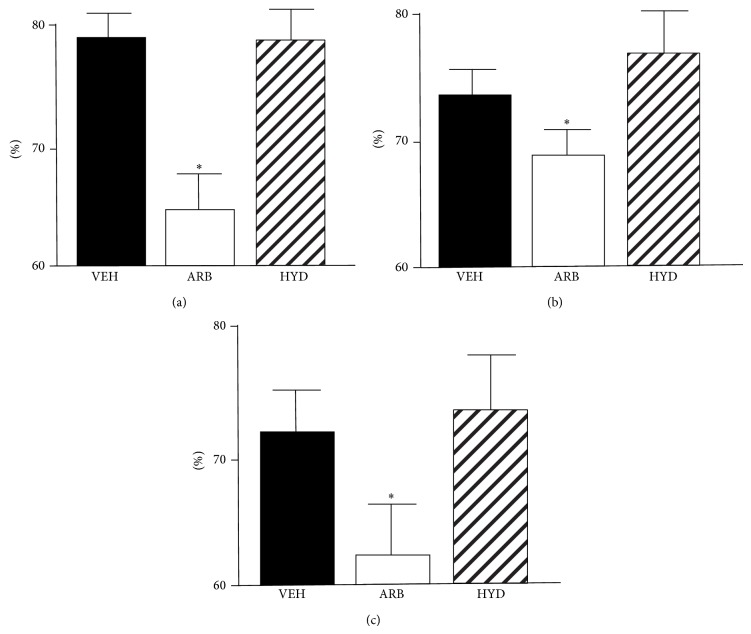
Normalized unit of the low-frequency component of systolic blood pressure as parameters *f* sympathoexcitation in stroke-prone spontaneously hypertensive rats treated with intracerebroventricular infusion of losartan (ARB, *n* = 5), oral-administered hydralazine (HYD, *n* = 5), and intracerebroventricular infusion of vehicle (VEH, *n* = 5) at early morning (a), after morning (b), and night (c). ^*^ *P* < 0.05 versus VEH.

LFnuSBP was shown in Figure 3. Throughout EM, AM, and NT, LFnuSBP was significantly lower in ARB than in HYD and VEH. In HYD, LFnuSBP did not differ compared to VEH.

### 3.3. Baroreflex Sensitivity

**Figure 4:**
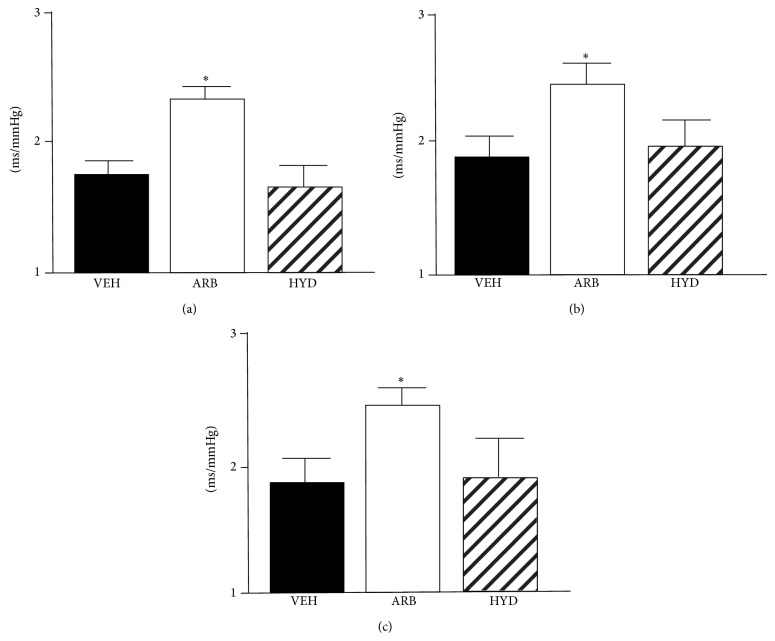
Baroreflex sensitivity calculated by spontaneous sequence method in stroke-prone spontaneously hypertensive rats treated with intracerebroventricular infusion of losartan (ARB, *n* = 5), oral-administered hydralazine (HYD, *n* = 5), and intracerebroventricular infusion of vehicle (VEH, *n* = 5) at early morning (a), after morning (b), and night (c). ^*^ *P* < 0.05 versus VEH.

Throughout EM, AM, and NT, baroreflex sensitivity was significantly higher in ARB than in HYD and VEH (Figure 4). In HYD, baroreflex sensitivity did not differ compared to VEH (Figure 4).

## 4. Discussion

Our obtained new findings were as follows. (1) At EM and NT, MBP was decreased in ARB to a greater extent than in HYD. (2) At AM, MBP was the same in ARB and HYD. (3) Throughout EM, AM, and NT, LFnuSBP was significantly lower in ARB than in VEH and HYD, and (4) baroreflex sensitivity was improved in ARB, but not in HYD. These results suggest that central AT_1_R blockade would attenuate the excess blood pressure elevation in early active phase and continuous blood pressure elevation during rest phase independent of depressor response in hypertension and that these benefits of central AT_1_R blockade on dairy blood pressure variability might be due to sympathoinhibition with baroreflex improvement.

The most impressive results were that central AT_1_R blockade attenuates the excess blood pressure elevation in early active phase. Dairy blood pressure variability and/or morning surge is associated with abnormal regulation of sympathetic nerve activity [9], and we have demonstrated that central AT_1_R blockade causes depressor response with sympathoinhibition [12, 17, 18]. Considering these backgrounds, our present results are reasonable. Moreover, in the present study, MBP at AM was the same in ARB and HYD, although MBP in HYD was significantly higher at EM and NT than at AM. MBP in VEH did not differ among EM, AM, and NT. We consider the blood pressure variability of VEH as “nondipper” type and that of HYD as “riser” type. These results strongly indicate that benefit of central AT_1_R blockade on MBP at EM was not due to depressor response itself. Interestingly, the benefit at EM was also determined during rest. In the clinical aspects, central AT_1_R blockade could archive dipper type dairy blood pressure variability in hypertension. We should assess dairy blood pressure variability, especially MBP at EM and NT, not only at active phase.

In the aspects of mechanisms, we also consider that central infusion of losartan could improve baroreflex sensitivity, resulting in the improvement of blood pressure variability. Previously we demonstrated that central infusion of AT_1_R blocker improved the impaired baroreflex sensitivity with sympathoinhibition and antioxidant effect in the brain of hypertensive rats [17]. AT_1_R-induced oxidative stress in the brain causes sympathoexcitation [17, 18], and reduction of central oxidative stress significantly improves baroreflex sensitivity in hypertensive rats [17, 20]. Considering those studies, AT_1_R-induced oxidative stress in the brain should worsen blood pressure variability via sympathoexcitation with baroreflex dysfunction. To determine these aspects, we calculated baroreflex sensitivity by spontaneous sequence method and demonstrated that baroreflex sensitivity was significantly higher in central losartan-treated SHRSP than in hydralazine- or vehicle-treated SHRSP throughout EM, AM, and NT. These results strongly support the conclusion that central AT_1_R blockade improves blood pressure variability via sympathoinhibition with improvement of baroreflex.

Although we showed that central application (intracerebroventricular infusion) of AT_1_R blocker is beneficial to abnormal blood pressure elevation, as previously shown in our and other works [17, 19], we had not determined angiotensin II content or AT_1_R expression in cardiovascular center and do not have the data of angiotensin II content and AT_1_R expression in RVLM in early morning, after morning, and night. Previous reports indicated that systemic circulatory and tissue renin-angiotensin system are significantly higher at active than at rest phase and that these abnormal circadian rhythms are attenuated by AT_1_R blocker [25–27]. Moreover, a previous study reported that central AT_1_R has circadian rhythm [28]. Considering these results, central infusion of losartan blocked AT_1_R in cardiovascular center strongly at angiotensin II-AT_1_R activated phase (night-active) and would improve the abnormal circadian rhythm of blood pressure. However, to assess more concrete mechanisms in which intracerebroventricular infusion of losartan improves blood pressure variability, it would be necessary in further examination to determine whether angiotensin II content and AT_1_R expression in cardiovascular center have circadian rhythm or not.

Our results proposed a novel clinical aspect. We had better focus on the central AT_1_R as the suitable target of the treatment with AT_1_R blockers. Recently, we have suggested that the beneficial effects on central AT_1_R were different among oral-administered AT_1_R blockers [18]. To archive the optimal quality and quantity of blood pressure in hypertension, we consider that it is preferable to use AT_1_R blockers affecting central AT_1_R.

## 5. Conclusions

Central AT_1_R blockade potentially attenuated excess blood pressure elevation in early active phase and continuous blood pressure elevation during rest phase via sympathoinhibition with improvement of baroreflex, independent of depressor response in hypertensive rats.
